# Lack of direct association between viral hepatitis and sleep disturbances

**DOI:** 10.3389/fmed.2022.951762

**Published:** 2022-11-14

**Authors:** Sheng-Jie Lin, Shang-Ching Joy Tang, Yu-Chia Lee, Ting-Yu Liu, Ting-Chun Huang, Rwei-Ling Yu, Chun-Hsiang Tan

**Affiliations:** ^1^School of Medicine, College of Medicine, Kaohsiung Medical University, Kaohsiung, Taiwan; ^2^Institute of Behavioral Medicine, College of Medicine, National Cheng Kung University, Tainan, Taiwan; ^3^Department of Neurology, Kaohsiung Medical University Hospital, Kaohsiung Medical University, Kaohsiung, Taiwan; ^4^Graduate Institute of Clinical Medicine, College of Medicine, Kaohsiung Medical University, Kaohsiung, Taiwan

**Keywords:** HBV, HCV, insomnia, sleepiness, sleep disorders

## Abstract

**Background:**

Individuals with chronic viral hepatitis are at increased risk of experiencing poor sleep quality and sleep disturbances. However, it remains unclear whether the sleep disorders associated with viral hepatitis are secondary to the comorbidities related to viral hepatitis or the direct effect of hepatitis viruses on sleep. This study investigated the direct impact of viral hepatitis B and C on sleep quality.

**Methods:**

Individuals with viral hepatitis B or C and their healthy counterparts were recruited for the present study, and they were evaluated with the Parkinson's Disease Sleep Scale-2, the Epworth Sleepiness Scale, and the Pittsburgh Sleep Quality Index in the absence of common comorbidities associated with viral hepatitis.

**Results:**

Neither hepatitis B nor hepatitis C was found to cause significant differences in insomnia symptoms or excessive daytime sleepiness. However, individuals with hepatitis C, but not hepatitis B, tended to be less likely to experience restlessness of the legs or arms at night.

**Conclusions:**

This study suggests that hepatitis viruses B and C may not cause a significant impact on sleep quality and related disorders directly. Sleep disturbances in individuals with chronic viral hepatitis may instead be attributable to hepatic decompensation or the comorbid factors associated with viral hepatitis.

## Introduction

Hepatitis, as an inflammation of the liver, can be self-limiting or can progress to severe complications such as cirrhosis or hepatocellular carcinoma (1). Globally, the leading cause of hepatitis is viral hepatitis. Among the five major hepatitis viruses, hepatitis B virus (HBV) and hepatitis C virus (HCV) infection frequently progress to chronic hepatitis. In addition to the hepatic complications, patients with HBV and HCV may also present with extrahepatic manifestations. These extrahepatic manifestations may not be lethal, but they can profoundly affect the quality of life (QOL) of the individuals infected. Furthermore, growing evidence has shown the detrimental impact of viral hepatitis on the central nervous system (CNS) functions, resulting in fatigue, sleep disturbance, lethargy, loss of appetite, depression, and anxiety (2, 3). Among the symptoms associated with CNS dysfunction, sleep disturbance may be particularly harmful, as evidenced by the strong association between sleep disturbance and the development of cardiovascular diseases, diabetes mellitus, obesity, and malignancy. Moreover, the diseases associated with sleep disturbance may reciprocally cause CNS dysfunction, resulting in a vicious cycle (4, 5).

Previous studies have reported an increased prevalence of sleep disturbances among patients with either HBV or HCV (6–9). Patients with chronic HCV were found to have a higher chance of experiencing poor sleep quality and sleep disturbances (10). Meanwhile, individuals with HBV-associated cirrhosis were reported to have a higher prevalence of sleep disturbance. Furthermore, the common complications among individuals with viral hepatitis, depression, and cirrhosis have been reported to be associated with sleep disturbances (11). Whether the higher prevalence of poor sleep quality and sleep disturbances observed in individuals with viral hepatitis is the result of the direct impact of hepatitis viruses on the CNS or secondary to the frequent comorbidities associated with the hepatitis virus infections remains undetermined or controversial. For example, one study showed that after controlling confounding factors, including depression, health perception, living status, and type of dwelling, no significant difference in insomnia was found between healthy controls and patients with hepatitis B (7).

As a result, to more clearly elucidate the direct impact of hepatitis B and C on sleep quality and associated disturbances, we set out to study the sleep profiles of individuals with HBV or HCV and their healthy counterparts in parallel while excluding individuals with decompensated hepatic cirrhosis, malignancy, or depression.

## Methods

### Participants and assessment of clinical presentations

The participants were recruited cross-sectionally from 2017 to 2020, aged between 40 and 80 years old. For hepatitis groups, we recruited patients positive for serum HBsAg or positive for serum anti-HCV IgG results. Among the 111 patients positive for anti-HCV IgG, 82 had obtained sustained virologic response (SVR) before being recruited. In addition, patients must have no other liver disease, no HBV and HCV co-infections, and no HIV co-infection. Age-matched healthy participants without HBV or HCV were recruited as the healthy control (HC) group. We excluded participants with signs suggestive of decompensated cirrhosis or a history of malignancy, end-stage renal disease, gastrointestinal variceal bleeding, substance abuse, alcohol consumption over 60 grams per day, uncontrolled psychiatric symptoms, or recreational drug use to exclude potential confounding factors.

Similar to our previous studies, written informed consent from the participants was obtained before enrollment, following the ethical standards outlined in the 1975 Declaration of Helsinki. All study procedures were approved by the ethical research committee of Kaohsiung Medical University Hospital and National Cheng Kung University Hospital. All methods were performed according to the approved guidelines.

Several evaluation tools, either subjective or objective, have been applied across different studies to assess sleep disorders and are widely used in medical practice. Nevertheless, there is still no consensus on a standardized evaluation protocol to determine the profiles or severity of sleep disorders among individuals with chronic liver disease. According to the International Classification of Sleep Disorders proposed by the American Academy of Sleep Medicine (12), sleep disorders can be categorized into seven major groups: insomnia, sleep-related breathing disorders, circadian rhythm sleep-wake disorders, parasomnias, sleep-related movement disorders, central disorders of hypersomnolence, and other sleep disorders. Among the different categories, insomnia, excessive daytime sleepiness (EDS), obstructive sleep apnea, and restless leg syndrome (RLS) are the most common among individuals with chronic liver disease. As a result, the sleep quality of the participants recruited in the present study was evaluated with Parkinson's Disease Sleep Scale-2 (PDSS-2), Epworth Sleepiness Scale (ESS), and Pittsburgh Sleep Quality Index (PSQI) (13).

### Questionnaires

The PSQI consists of 19 self-rated questions regarding subjects' sleeping habits and five questions intended for the subject's sleeping partner and was used to assess the sleep quality and disorders of the participants over the past month (14). The self-rated questions were used to calculate seven component scores, including subjective sleep quality, sleep latency, sleep duration, habitual sleep efficiency, sleep disturbances, use of sleeping medication, and daytime dysfunction. The seven component scores were summed up to produce a global score. Each component score ranges from 0 to 3, with a higher score indicating more difficulty. The global PSQI score ranges from 0 to 21, and a score higher than 5 indicates poor overall sleep quality.

The PDSS-2, created initially to quantify the different aspects of nocturnal sleep problems in Parkinson's disease, was also used to evaluate the quality of night sleep, sleep onset, maintenance insomnia, nocturnal restlessness and psychosis, nocturia, nocturnal motor symptoms, sleep refreshment, and dozing in the daytime of the participants among the different study group in this study. Each component score signifies the frequency of the symptoms and ranges from 0 to 4, with four meaning 6–7 days a week and zero meaning never (15, 16).

The ESS was used to assess daytime sleepiness symptoms under eight different situations over the past month (17). The participants were asked to grade their chances of dozing off from a range of 0–3 by themselves. The eight component scores were then summed up to produce a global score representing daytime sleepiness. The global score ranges from 0 to 24, and an ESS score higher than eight indicates daytime sleepiness disorder. A higher global score indicates a more severe daytime sleepiness disorder.

### Statistical analysis

Similar to our previous studies, proportions were calculated for qualitative variables, and means and standard deviations (SDs) were calculated for quantitative variables (18). The data were examined for normality and homogeneity of variance. Afterward, we tested quantitative variables with the two-sample *t*-test, Kruskal Wallis test, one-way ANOVA, or Mann-Whitney U test, and qualitative variables using a chi-square test. For the comparisons of the items in the sleep questionnaires, the non-parametric variables, between the different study groups, we used the Kruskal-Wallis test to screen for statistical significance. For the items with a *p*-value lower than 0.05 in the Kruskal-Wallis test, we performed the *post-hoc* analysis with Quade's test to control the impact attributable to confounding variables (age, sex, and presence of hypertension, diabetes mellitus, and hyperlipidemia). As metabolic syndromes have been shown to contribute to poor sleep quality (19–30), we set the presence of hypertension, diabetes mellitus, and hyperlipidemia, in addition to age and sex, as covariates to minimize the potential confounding effect secondary to the differences in the potential confounding factors. The statistical significance level for demographic data was set at the conventional level of 0.05, while the statistical significance level for the items in the sleep disorder questionnaires was determined with Bonferroni correction by dividing 0.05 by the number of items compared across the three questionnaires, 32, to obtain a cut-off value of 0.00156. A commercially available software program performed the statistical analysis (IBM Corp. Released 2012. IBM SPSS Statistics for Windows, Version 25.0. Armonk, NY: IBM Corp.). All the associated data not provided within the paper are available on request from Chun-Hsiang Tan.

## Results

### Demographic and clinical characteristics

A total of 291 participants between 40 and 80 years of age were recruited, including 105 participants as healthy controls (HCs), 75 patients with HBV, 29 patients with treatment-naïve HCV, and 82 patients with treatment-experienced HCV. Among the 291 participants, 195 participants completed PDSS-2 (99 HCs, 74 patients with HBV, and 22 patients with HCV), 263 participants completed the PSQI (91 HCs, 70 patients with HBV, and 102 patients with HCV), and 195 participants completed the ESS (99 HCs, 74 patients with HBV and 22 patients with HCV). The demographic characteristics are shown in Table 1. The mean age of HCs, patients with HBV, patients with treatment-experienced HCV, and patients with treatment-naïve HCV were 61.56 ± 5.809, 59.33 ± 7.076, 61.95 ± 9.269 years, and 61.66± 8.010 years, respectively (*p* = 0.122). Among the participants, 40.0% were male, including 23.8% in the control group, 70.7% in the HBV group, 41.5% in the treatment-experienced HCV group, and 13.8% in the treatment-naïve HCV. The percentage of the participants being male was significantly higher in the hepatitis B group (*p* < 0.001) compared to other groups. In addition, the percentages of individuals with hypertension in HCs, HBV, treatment-experienced HCV, and treatment-naïve HCV groups were 16.2, 33.3, 37.8, and 34.5%, while the percentages of individuals with diabetes mellitus in HCs, HBV, treatment-experienced HCV, and treatment-naïve HCV groups were 7.6, 13.3, 22, and 10.3%. There were significant differences in the percentages of individuals with hypertension and diabetes mellitus among the three groups. In contrast, no statistically significant differences in the percentages of individuals with hyperlipidemia were observed between the study groups. The alanine transaminase (ALT) levels of patients with HBV, treatment-experienced, and treatment-naïve HCV were 29.49 ± 19.85 units/L, 24.99 ± 11.67 units/L, and 65.14 ± 57.60 units/L, respectively (*p* < 0.001), with the ALT level to be significantly higher in the individuals with treatment-naïve HCV.

**Table 1:** Demographic and clinical characteristics of the study groups.

	**HC (*n* = 105)**	**HBV (*n* = 75)**	**treatment-experienced HCV (*n* = 82)**	**treatment-naïve HCV (*n* = 29)**	**Statistic**	***p*-value**
	**Mean ±SD**	**Mean ±SD**	**Mean ±SD**	**Mean ±SD**		
Age (years)	61.56 ± 5.809	59.33 ± 7.076	61.95 ± 9.269	61.66 ± 8.010	*F*_(3, 287)_ = 1.946	0.122
Male (%)	25 (23.8)	53 (70.7)	34 (41.5)	4 (13.8)	χ^2^ = 49.284	< 0.001^*^
Hypertension (%)	17 (16.2)	25 (33.3)	31 (37.8)	10 (34.5)	χ^2^ = 12.655	0.005^*^
Hyperlipidemia (%)	19 (18.1)	22 (29.3)	25 (30.5)	7 (24.1)	χ^2^ = 4.738	0.193
Diabetes mellitus (%)	8 (7.6)	10 (13.3)	18 (22)	3 (10.3)	χ^2^ = 8.423	0.038^*^
ALT (U/L)	-	29.49 ± 19.85	24.99 ± 11.67	65.14 ± 57.60	*F*_(2, 173)_ = 23.816	< 0.001^*^

HBV, hepatitis B virus; HCV, hepatitis C virus; HC, healthy control; SD, standard deviation.

* Indicates *p*-value < 0.05.

To understand the impact of hepatitis viruses on sleep quality and related disorders, we examined whether a significant difference in sleep quality and sleep disorders existed between the three groups of participants based on the participants' responses on PSQI, PDSS-2, and ESS. By comparing the results of each item in the PSQI, as shown in Table 2, with the Kruskal-Wallis test, we found a borderline difference in the degree of daytime dysfunction (H = 6.221, *p* = 0.045) among the study groups. We then used Quade's test to perform a *post-hoc* analysis to compare the results between the three study groups and set age, sex, and the presence of hypertension, diabetes mellitus, and hyperlipidemia as covariates to eliminate the potential effect secondary to the confounding factors. However, with Quade's test, no statistically significant difference was observed in the level of daytime dysfunction among the study groups. These results indicate that HBV or HCV may not directly influence the severity of daytime dysfunction.

**Table 2:** The evaluation of sleep disturbance profiles with PSQI between different study groups.

	**Healthy control** **(*****n*** = **91)**		**Hepatitis B (*****n*** = **70)**		**Hepatitis C (*****n*** = **102)**		**Kruskal-Wallis test**		**Quade's test^+^**	
	**Mean**	**SD**	**Mean**	**SD**	**Mean**	**SD**	**Kruskal-Wallis H**	***p*-value**	**Group pairs**	
Subjective sleep quality	1.14	0.783	1.24	0.788	1.25	0.898	0.888	0.641	-	
Sleep latency	0.92	0.885	1.14	0.967	1.25	1.085	4.165	0.125	-	
Sleep duration	1.30	0.810	1.27	0.760	1.23	1.004	0.555	0.758	-	
Habitual sleep efficiency	0.54	0.970	0.50	0.881	0.64	0.993	1.174	0.556	-	
Sleep disturbances	1.09	0.486	1.14	0.490	1.16	0.502	1.147	0.564	-	
Use of sleeping medication	0.34	0.885	0.46	1.003	0.71	1.232	5.030	0.081	-	
Daytime	0.43	0.669	0.46	0.582	0.29	0.607	6.221	0.045^*^	HC-HBV	0.870
dysfunction									HC-HCV	0.253
									HBV-HCV	0.117
Global PSQI score	5.76	3.532	6.21	3.538	6.52	3.770	2.765	0.251	-	

+ Quade's test (age, sex, hypertension, diabetes mellitus, hyperlipidemia were set as covariates).

HBV, hepatitis B virus; HCV, hepatitis C virus; HC, healthy control; SD, standard deviation.

* Indicates *p*-value < 0.05.

We made further analysis by dividing patients with HCV into two groups according to whether they had obtained sustained virological response after treatment against HCV. However, due to the small sample number of participants completing ESS and PDSS-2 questionnaires, we could only compare the PSQI results of these two groups, as shown in Table 3. Using the Mann-Whitney U test, a borderline difference between the two groups was found in the degree of habitual sleep efficiency (U = 745.5, *p* = 0.018). With Quade's test, treatment-naïve patients showed the tendency to have better sleep efficiency than their counterparts who have obtained sustained virological responses (*p* = 0.028).

**Table 3:** The evaluation of sleep disturbance profiles with PSQI between treatment-naïve and treatment-experienced patients with HCV.

	**Treatment-naïve** **(*****n*** = **27)**		**Treatment-experienced** **(*****n*** = **75)**		**Mann-Whitney U test**		**Quade's test^+^**
	**Mean**	**SD**	**Mean**	**SD**	**U**	***p*-value**	***p*-value**
Subjective sleep quality	1.44	0.974	1.19	0.865	851	0.192	-
Sleep latency	1.44	1.086	1.17	1.083	865.5	0.246	-
Sleep duration	1.22	1.086	1.23	0.981	1,001.5	0.931	-
Habitual sleep efficiency	0.26	0.656	0.77	1.06	745.5	0.018^*^	0.028^*^
Sleep disturbances	1.26	0.656	1.12	0.434	903	0.268	-
Use of sleeping medication	0.63	1.214	0.73	1.245	960	0.605	-
Daytime dysfunction	0.26	0.526	0.31	0.636	1,001	0.905	
Global PSQI score	6.52	3.577	6.52	3.86	960	0.689	-

+ Quade's test (age, sex, hypertension, diabetes mellitus, hyperlipidemia were set as covariates).

HCV, hepatitis C virus; SD, standard deviation.

* Indicates *p*-value < 0.05.

### Individuals with hepatitis C showed a tendency for less severe “nocturnal restlessness of legs or arms”

Comparing the results of the PDSS-2 items with the Kruskal-Wallis test, we found a borderline difference between-group differences in the “maintenance insomnia” (*p* = 0.047) and “nocturnal restlessness of legs or arms” (*p* = 0.030) among the study groups, as shown in Table 4. With the Quade's test to compare the item scores of the group pairs in the “maintenance insomnia” and “nocturnal restlessness of legs or arms,” only a marginally significant difference in the “nocturnal restlessness of legs or arms” between the participants in the HCV and HC group (HC vs. HCV: *p* = 0.002) was observed. None of the participants in the HCV group reported “nocturnal restlessness of legs or arms,” while the mean score reported by the HC group was 0.47 ± 0.993. These results suggest that individuals with HCV may have the trend for less severe symptoms in “nocturnal restlessness of legs or arms”.

**Table 4:** The evaluation of sleep disturbance profiles with PDSS2 between different study groups.

	**Healthy control** **(*****n*** = **99)**		**Hepatitis B (*****n*** = **74)**		**Hepatitis C (*****n*** = **22)**		**Kruskal-Wallis test**	**Quade's test^+^**	
	**Mean**	**SD**	**Mean**	**SD**	**Mean**	**SD**	***p*-value**	***p*** **-value**	
All quality of night's sleep	1.07	1.296	0.97	1.227	0.59	1.098	0.215	
Sleep onset	0.81	1.113	0.97	1.324	1.04	1.430	0.843	
								HC-HBV	0.528
Maintenance insomnia	0.79	1.118	0.87	1.260	0.22	0.528	0.047^*^	HC-HCV	0.039^*^
								HBV-HCV	0.016^*^
Nocturnal restlessness of	0.47	0.993	0.35	0.867	0.00	0.000	0.030^*^	HC-HBV	0.170
legs or arms								HC-HCV	0.002^#^
								HBV-HCV	0.076
Fidget in bed	0.42	0.882	0.41	0.936	0.04	0.213	0.094	
Distressing dream	0.36	0.788	0.47	1.023	0.31	0.894	0.798	
Distressing hallucination	0.16	0.724	0.67	0.478	0.00	0.000	0.321	
Unable to turn around in bed or move due to immobility	2.28	1.443	2.58	1.261	2.22	1.572	0.366	
Wake up at night due to painful arms and legs	0.25	0.747	0.25	0.741	0.00	0.000	0.163	
Wake up at night due to muscle cramp in patients' arms and legs	0.35	0.837	0.43	1.008	0.45	0.213	0.143	
Wake up at night due to muscle cramps in patients' arms and legs	0.41	0.833	0.37	0.735	0.36	0.658	0.999	
Wake up early due to painful arms and legs in the morning	0.33	0.857	0.35	0.867	0.00	0.000	0.067	
Tremor on waking	0.14	0.700	0.08	0.490	0.00	0.000	0.557	
Sleep refreshment	0.80	1.143	0.86	0.926	0.45	1.011	0.058	
Shortness of breath at night	0.18	0.719	0.24	0.679	0.00	0.000	0.079	

+ Quade's test (age, sex, hypertension, diabetes mellitus, hyperlipidemia were set as covariates).

# indicates the *p*-value close to the significance cut-off value determined by the Bonferroni correction.

HBV, hepatitis B virus; HCV, hepatitis C virus; HC, healthy control; SD, standard deviation.

* Indicates *p*-value < 0.05.

Meanwhile, as shown in Table 5, we found no significant difference in the ESS score among the different study groups, indicating that viral hepatitis does not cause severe daytime sleepiness after eliminating the potential confounding factors.

**Table 5:** The evaluation of sleep disturbance profiles with ESS between different study groups.

	**Healthy control** **(*****n*** = **99)**		**Hepatitis B** **(*****n*** = **74)**		**Hepatitis C** **(*****n*** = **22)**		**Kruskal-Wallis test**	
**ESS items**	**Mean**	**SD**	**Mean**	**SD**	**Mean**	**SD**	**Kruskal-Wallis H**	***p*-value**
Sitting and reading	0.59	0.769	0.77	0.915	0.45	0.800	0.221	0.365
Watching TV	0.71	0.811	0.77	0.869	0.86	1.082	0.914	0.945
Sitting inactively in a public place	0.45	0.773	0.46	0.686	0.36	0.658	0.776	0.937
As a passenger in a car	0.94	1.038	0.82	0.942	0.82	0.858	0.779	0.622
Lying down to rest in the afternoon	1.08	1.131	1.26	1.061	1.27	1.120	0.497	0.884
Sitting and talking to someone	0.11	0.347	0.22	0.556	0.27	0.703	0.313	0.327
Sitting quietly after lunch	0.77	0.978	0.81	0.961	0.73	1.120	0.745	0.789
In a car while stopped	0.18	0.413	0.18	0.449	0.18	0.395	0.908	0.395
ESS total score	4.828	3.944	5.284	4.381	4.955	4.380	0.914	0.918

## Discussion

Individuals with chronic liver disease, such as fatty liver and liver cirrhosis, have been reported to have a higher risk for sleep disorders, even in the absence of neuropsychiatric problems (31–35). Among the various etiologies leading to chronic liver disease, viral hepatitis has always been the major cause (36). Meanwhile, viral hepatitis has also been shown to cause an increased risk for sleep disorders (6–9). Furthermore, as the comorbidities closely associated with chronic liver diseases, such as diabetes mellitus, hypertension, depression, and cardiovascular diseases (37–42), may by themselves cause impairment in the quality and duration of sleep, the impact of various etiologies or medical conditions contributing to the development of sleep disorders in individuals with chronic liver disease warrants further investigation. In this study, we set out to elucidate the direct impact of viral hepatitis on the development of sleep disorders.

Previous studies evaluating the prevalence of sleep disorders in patients with chronic liver disease mainly focused on patients with cirrhosis. One previous study reported the prevalence of sleep disorders to be 47.7% in patients with cirrhosis, while another study reported that up to 69% of patients with cirrhosis complained of sleep problems and depression (43, 44). Due to the difference in the assessment tools applied and the populations recruited in each study, the prevalence of sleep disorders diverges significantly. Apart from the reasons above, differences in individual characteristics, such as age, gender, race, and comorbidities, may also cause differences in the prevalence of sleep disorders. As a result, these factors were set as covariates in the analyses in the present study. More importantly, patients with severely decompensated cirrhosis and depression were excluded during the recruitment to elucidate the direct impact of viral hepatitis B and C on sleep quality since both decompensated cirrhosis and depression profoundly affect sleep.

Insomnia is prevalent among individuals with chronic liver disease (45, 46), and about 42–65% of individuals with cirrhosis were estimated to suffer from insomnia (47). In the present study, 48.57, 52.94, and 43.96% of patients with HBV, HCV, and healthy controls were found to be poor sleepers (defined as PSQI global score >5). However, though a higher proportion of patients with chronic viral hepatitis was reported to have sleep impairment, we found no statistically significant differences in the severity of insomnia symptoms between these patients and their healthy counterparts. Our findings imply that hepatic viruses *per se* may not directly influence the extent of insomnia symptoms and sleep disturbances, compatible with the results suggesting that sleep impairment in chronic liver disease corresponds to disease severity and the degree of hepatic decompensation. A previous case-control study noted a significant correlation between insomnia symptoms and HBV disease severity in 120 patients with HBV-related diseases (7). Additionally, sleep disturbances may be associated with somatic complaints experienced by patients with advanced liver cirrhosis. One study noted that muscle cramps, a complication of end-stage liver disease, were independently associated with disturbed sleep (48). Furthermore, hyperammonemia, which is strongly associated with the severity of hepatic decompensation, has also been shown to be an important contributor to sleep disturbances among patients with cirrhosis (49). However, it remains controversial whether sleep impairment in patients with chronic liver disease is associated with the presence of hepatic encephalopathy (HE). For example, no significant association between insomnia and psychometric performance was found in a study recruiting 44 patients with cirrhosis (among whom 24 had minimal HE) (43), while another study recruiting 87 patients with cirrhosis found no association between insomnia and the presence or severity of HE (31). In contrast, a case-control study found that in 178 patients with hepatic cirrhosis, those with minimal HE experienced significantly more frequent insomnia (46). Similarly, a notable correlation between poor cognition and poor sleep quality was observed among 100 patients with cirrhosis (50), and a cohort study evaluating 341 patients with hepatitis B-related liver cirrhosis found that PSQI scores were markedly higher in patients with minimal HE (8). Overall, there is diverging evidence regarding the relationship between sleep impairment and hepatic encephalopathy. Nevertheless, as the patients with HE were excluded from the present study, only subjects with minimal liver disease who had yet to progress to hepatic decompensation were included in our patient groups. The lack of direct impact of viral hepatitis on sleep disturbances observed in the present study may also support the idea that insomnia and sleep disturbances observed in individuals with chronic liver disease may be more strongly correlated with liver disease severity and hepatic decompensation.

Several studies have reported EDS as one of the primary complaints of individuals with chronic liver disease, besides insomnia. Previous studies have also found that around 21–50% of individuals with cirrhosis suffer from sleepiness disorders (33, 46). Besides, in individuals with chronic liver disease, EDS was found to be correlated with significant electroencephalographic slowing and both the history and development of HE (31, 51). Although one study found no correlation between EDS and HE, they also observed that significant daytime sleepiness was associated with the slowing of the EEG mean frequency (31). Overall, the studies shown above favored a strong association between EDS and HE. By excluding the participants with decompensated cirrhosis or active neuropsychiatric symptoms, the present study found no significant difference in the symptoms of EDS between the individuals with chronic viral hepatitis and their healthy counterparts when assessed with ESS or the associated items in PSQI. Our results suggest that viral hepatitis, either HBV or HCV, does not cause significant EDS symptoms before the progression to decompensated cirrhosis. The EDS observed previously in individuals with chronic liver disease may be secondary to HE development, as evidenced by the association between EDS and hepatic decompensation.

RLS was reported to be prevalent among patients with chronic liver disease and is considered one of the causes of sleep disturbance. For example, one study found a high prevalence rate of RLS, up to 62% among 141 individuals with chronic liver disease, while the prevalence rate of RLS among the general population was 10% (52). Besides, one Japanese study screened for the presence of RLS among 149 individuals with chronic liver disease (53). The study found a prevalence rate of 16.8%, higher than that in the general Japanese population (53, 54). As cirrhosis is associated with sleep disturbance, several studies explored the association between cirrhosis and RLS (43, 52). One study showed a high prevalence of RLS (26.11%) among cirrhotic subjects (55), while another study found a poor correlation between liver disease severity and the presence of RLS in patients with cirrhosis (56). Even though several studies have investigated whether RLS mediates the association between sleep disorders, chronic liver disease, and cirrhosis, few studies have focused on the difference in RLS prevalence between individuals with different hepatitis viruses and their healthy control counterparts. In this study, the mean score of “nocturnal restlessness of legs or arms” in PDSS-2 in the study group of HBV, HCV, and healthy controls were 0.35, 0.00, and 0.47, respectively, and a marginally significant difference was found between individuals with HCV and the healthy control group. The finding suggests that individuals with HCV may have the trend for less severe “nocturnal restlessness of legs or arms” than those in the healthy control group.

There could be several explanations for the unexpected trend. First, in the study reporting the high prevalence of RLS at 62% among the patients with chronic liver disease, several medical conditions that are strongly associated with chronic liver diseases and predisposing to the development of RLS, such as end-stage renal diseases, metabolic syndromes (57, 58), or depression (59), were not excluded or controlled (52). Similarly, in the Japanese study mentioned above, the potential effect of other risk factors was not controlled, either (53). On the other hand, these confounding factors were either excluded or adjusted in the present study. More importantly, among the studies investigating the prevalence of RLS among individuals with chronic liver disease, the impact of viral hepatitis, either HBV or HCV, has never been explicitly evaluated. However, the present study found the trend that individuals with HCV tended to report less severe “nocturnal restlessness of legs or arms” than healthy control counterparts after excluding common risk factors associated with RLS. Further investigation may be needed to validate the observation and explore for the mechanism underlying the phenomenon.

In addition, after taking into account the influence of confounding factors, we found the trend that the treatment-naïve patients in the HCV group reported better sleep efficiency than the treated patients. As our patients with HCV were either treated with interferon or direct-acting antiviral (DAA), both of which had a direct impact on sleep (60–62), the finding may suggest that the adverse effects of treatment against HCV may last long after the patients achieving sustained virological responses, resulting in inefficient sleep. The result also supports our conclusion that viral hepatitis *per se* does not cause a direct impact on sleep disturbances. Those sleep disorders observed in individuals with chronic liver disease may be secondary to hepatic decompensation or the effect of the medications.

The present study has potential limitations. First, only a part of the participants fulfilling the inclusion criteria had the patience to complete all the questions listed on the three questionnaires. Hence, we regret to find that a relatively small number of patients had completed the PDSS-2 and the ESS. The uneven number of participants completing the various evaluation tools in the different participant groups may limit the power to detect certain aspects of sleep disorders. For example, for the excessive sleepiness symptoms evaluated with the ESS, the number of participants with HCV (*n* = 22) completing the ESS questionnaire was relatively small and may reduce the sensitivity in detecting the subtle difference between the different study groups. Second, although we excluded or controlled several factors to eliminate confounding effects in the present study, there were still some issues that we did not control. For instance, sleep medication usage, genetic heredity, and social stress were hard to research with questionnaires thoroughly. Since our questionnaires were already too long and complicated for participants to complete patiently, it is not suitable for us to add more questions and affect our sample size. Finally, since our current study is based on questionnaires, the disadvantages secondary to the nature of questionnaire-based assessment, such as recall bias, may not be avoided. Future studies with larger sample sizes, more detailed participant information, and other methods to measure sleep profiles objectively may be needed to validate the results of this study.

In conclusion, to elucidate the direct impact of hepatitis B and hepatitis C on the development of sleep disturbances, we used PSQI, PDSS-2, and ESS to explore sleep profiles among patients with viral hepatitis and healthy participants. After excluding psychiatric disorders, decompensated liver cirrhosis, substance abuse, and other conditions that might impact sleep disturbance, our results do not support a significant effect of HBV or HCV on the development of insomnia or excessive daytime sleepiness. The lack of association between viral hepatitis and sleep disturbances in the absence of severe hepatic decompensation or comorbidities may suggest that the development of sleep disturbances depends on hepatic decompensation or comorbidities associated with chronic liver disease. In addition, patients with HCV seemed to show a tendency toward less severe RLS symptoms than their healthy control counterparts. These results facilitate a better understanding of the effect of viral hepatitis on sleep disorders, and future studies with a larger sample size applying quantitative measures may be warranted to validate the results of this study with the hope of developing better strategies for the treatment of sleep disorders frequently observed in individuals with chronic liver disease.

## Data availability statement

All the associated data not provided within the paper are available on reasonable request from C-HT.

## Ethics statement

Written informed consent from the participants was obtained before enrollment. All study procedures were approved by the Ethical Research Committee of Kaohsiung Medical University Hospital and National Cheng Kung University Hospital. All methods were performed according to the approved guidelines.

## Author contributions

C-HT and R-LY had the idea and designed the experiments and were involved in data collection. S-JL, S-CT, Y-CL, T-YL, and T-CH performed data analysis. S-JL, S-CT, Y-CL, T-YL, T-CH, and C-HT wrote the paper and took responsibility for interpreting the results. C-HT accepted full responsibility for the work and controlled the decision to publish. All authors critically reviewed drafts and approved the final version of this article, fulfilled the authorship criteria, and no one who met the criteria was excluded.

## Funding

This study was supported by grants from the National Health Research Institutes, Taiwan (NHRI-EX111-11115NC), Ministry of Science and Technology, Taiwan (MOST 108-2320-B-037-034-MY3 and MOST 110-2628-B-006-020-), and Kaohsiung Medical University (KMU-DK107010).

## Conflict of interest

The authors declare that the research was conducted in the absence of any commercial or financial relationships that could be construed as a potential conflict of interest.

## Publisher's note

All claims expressed in this article are solely those of the authors and do not necessarily represent those of their affiliated organizations, or those of the publisher, the editors and the reviewers. Any product that may be evaluated in this article, or claim that may be made by its manufacturer, is not guaranteed or endorsed by the publisher.
